# Amyloidosis due to TTR mutations in Mexico with 4 distincts genotypes in the index cases

**DOI:** 10.1186/s13023-018-0801-y

**Published:** 2018-07-03

**Authors:** Alejandra González-Duarte, Karla Cárdenas-Soto, Carlo Enrico Bañuelos, Omar Fueyo, Carolina Dominguez, Benjamín Torres, Carlos Cantú-Brito

**Affiliations:** Department of Neurology and Psychiatry, Instituto Nacional de Ciencias Médicas y Nutrición Salvador Zubirán, Vasco de Quiroga 15 Col. Belisario Dominugez Sección XVI, CP 14080 Tlalpan, CDMX Mexico

**Keywords:** Hereditary amyloidosis, TTR Ser50Arg, Ser52Pro, Gly47Ala mutations

## Abstract

**Background:**

Transthyretin-related hereditary amyloidosis (ATTR) is a systemic disease characterized by extracellular deposits of amyloid due to the autosomal dominant inheritance of a mutation in the TTR gene (18q12.1). Although described worldwide, it is a rare disease, limited to certain parts of the world.

The aim of this manuscript is to describe the presence and characteristics of ATTR in Mexico.

**Methods:**

From 2010 through the database of the Instituto Nacional de Ciencias Médicas y Nutrición Salvador Zubirán we selected cases that had the diagnosis of “amyloidosis”, with any etiology, reviewed the files and selected those with suspected hereditary etiology.

**Results:**

We identified 111 subjects with 5 different pathological mutations, none of them with the classic Val30Met mutation. Mutations found were Ser50Arg in 83 (74%), Gly47Ala in 14 (13%), Ser52Pro in 12 (11%) and V122I /Y116H in 2 (2%). The majority of positive patients were from the States of Morelos and Guerrero. Twenty different families were included. The most common causes of death was urosepsis and cardiac failure.

**Conclusions:**

In Mexico there are endemic foci of ATTR, mainly in the states of Morelos and Guerrero. The major mutations are different from the most common global mutation Met30Val.

## Background

Amyloidoses are systemic diseases characterized by extracellular deposits of beta-folded proteins [1]. The most common inherited form is transthyretin-associated amyloidosis (ATTR), previously known as familial amyloid polyneuropathy (PAF) or Portuguese amyloidosis [1, 2]. This disease is associated with mutations in the transthyretin gene (TTR), it presents with progressive systemic alterations and has a fatal outcome between 30 and 40 years of age [3]. It is a rare disease with an uneven distribution around the world. Initially it was described for the first time in Portugal and later in other countries as endemic foci, particularly in Japan and Sweden [1–3].

Transthyretin is a tetrameric protein composed of 127 amino acids synthesized in the liver [1, 2]. TTR gene alterations cause a dissociation of the tetramer into monomers, forming amyloid deposits in several organs [3]. There are more than 100 mutations of the TTR gene, which are transmitted in an autosomal dominant manner and result in variable phenotypic expressions [4]. The most common mutation at the global level is the Val30Met that causes the classic picture of peripheral polyneuropathy.

The clinical sign is characterized by sensory and progressive motor polyneuropathy that begins to manifest from the third and fourth decade of life and that causes disability and death in a few years. All forms of amyloidosis associated with the TTR gene are progressive, encompassing a variable life expectancy that is thought to be dependent on the mutation of the gene, although certain factors have been described that modify its expression [4, 5]. The large number of pathological changes associated with this small gene suggest a delicate conformation extremely susceptible to disruption to any changes occurring in any of its structures [6]. The presentation of the disease and the age of onset of symptoms depend directly on environmental factors, from the place of origin and expression of the gene, to the TTR mutation itself [4, 5]. Late-onset cases with distinct clinicopathological features, even among same mutation, are prevalent in non-endemic areas [7]. The symptomatology, clinical course, penetrance and age of onset of ATTR carriers vary even in patients with the same mutation, and depend to a great extent on the geographical region and the type of population [5–7].

We previously described the clinical characteristics of some of the patients with S50A, S52P and G47A mutations [8, 9]. The aim of this manuscript is to broaden the information regarding the place of origin and genealogies, and to estimate the prevalence of the disease in a latinamerican country.

## Methods

A genetic study was carried out on 11 selected cases from the database with the diagnosis of amyloidosis, 62 first-degree relatives at risk, and 64 patients referred by the outpatient clinic and other institutions with suspected hereditary amyloidosis at the National Institute of Sciences Medical and Nutrition, national tertiary reference center. After discarding non-hereditary causes of amyloidosis, the informed consent was signed and the symptom questionnaire was aswered, the genetic test was performed using a blood sample (3 tests) and saliva (211 tests) and sent to different commercial laboratories abroad (Ambry Genetics, Mayo Clinic and Genos Medica) for analysis of the complete sequence of the TTR gene. Variations of the DNA sequence were identified by automated direct sequencing. Exons 1 through 4 and at least 20 bases were analyzed toward the 5 ‘and 3’ ends of all introns. The database was used *Human Gene Mutation Database* (http://www.hgmd.cf.ac.uk) to compare our results with the mutations and polymorphisms previously described.

The screening questionnaire for both patients and relatives included specific symptomatology at the sensory, motor, cardiovascular, digestive, genitourinary and autonomic levels. The neurological examination included assessment of muscle strength, muscle stretching reflexes, and the sensitive part (pain, fine touch and temperature sensation), as well as orthostatic blood pressure (decubitus and after three minuts of standing). Other pathologies associated with amyloidosis, such as hematological disorders (multiple myeloma), rheumatologic disorders (rheumatoid arthritis), or infectious diseases (tuberculosis or HIV) were ruled out. Once the positive results were obtained, the diagnostic work out was completed with a family history, serum studies, nerve conduction velocities, echocardiogram, electrocardiogram, cardiovascular autonomic tests, bladder ultrasound, and biopsies. We defined that patients had ATTR when they had evidence of amyloid deposits obtained by Congo Red staining positive in at least one affected organ (periumbilical fat, intestine, peripheral nerve or muscle), signs and symptoms compatible with the disease and mutations in the TTR gene [10]. Patients without symptoms with positive mutations were classified as asymptomatic carriers.

Our study was approved by the Research Ethics Committee of the Instituto Nacional de Ciencias Médicas y Nutrición Salvador Zubirán (INCMNSZ). All subjects signed an informed consent letter prior to the completion of the genetic study. We used the SPSS 16.0 database for statistical analysis.

## Results

Since 2010, 214 genetic tests have been performed in search of mutations in the TTR gene with the suspicion of hereditary amyloidosis. Of these, 97 (45%) were men and 117 (54%) were women. The median age was 37 years, with a range between 18 and 77 years of age. One hundred eleven (52%) of the tests were positive for at least one mutation (two samples had a heterozygous mutation). The mutations found were Ser50Arg in 83 (74%) patients, Gly47Ala in 14 (13%) patients, Ser52Pro in 12 (11%) patients and two (2%) patients, father and daughter, with heterozygous V122I /Y116H mutations (Table 1).

**Table 1:** Description of the main mutations

	Ser50Arg *n* = 83	Gly47Ala *n* = 14	Ser52Pro *n* = 12	V122I /Y116H *n* = 2
Female gender	44 (53%)	9 (64%)	5 (42%)	1 (50%)
Age	35 ± 12 (18-77)	36 ± 12.2 (19-55)	33 ± 13.9 (20-59)	62 ± 16.9 (50-74)
Place of Origin Guanajuato	0	0	12 (100%)	0
Guerrero	39 (47%)	5 (36%)	0	2 (100%)
Morelos	32 (39%)	8 (57%)	0	0
Ciudad de México	12 (14%)	0	0	0
Other states	0	1 (7%)	0	0
Number of Pedigrees	16	2	1	1
Age of onset of symptoms	35 ± 10	35 ± 14	36 ± 14	65
Symptomatic patients	52 (62%)	7 (50%)	6 (50%)	1 (50%)
Initial symptom neurological	34 (65%)	5 (71%)	5 (83%)	1 (100%)
Autonomic	6 (12%)	0	1 (17%)	0
Cardiological	3 (6%)	0	0	0
Gastrointestinal	9 (17%)	2 (29%)	0	0
Ophthalmologic		0	0	0
Difficulty walking at the time of study	17 (20%)	3 (21%)	3 (25%)	0
Clinical Stage PNP to the admission				
Stage 0	32 (38%)	7 (50%)	7 (58%)	0
Stage 1	20 (24%)	3 (21%)	2 (17%)	1 (50%)
Stage 2	25 (30%)	2 (14%)	2 (17%)	0
Stage 3a	4 (5%)	0	1 (8%)	0
Stage 4	2 (2%)	2 (14%)	0	1 (50%)
Death during the study	8 (10%)	1 (7%)	2 (17%)	0

### Source of reference

**Fig. 1:**
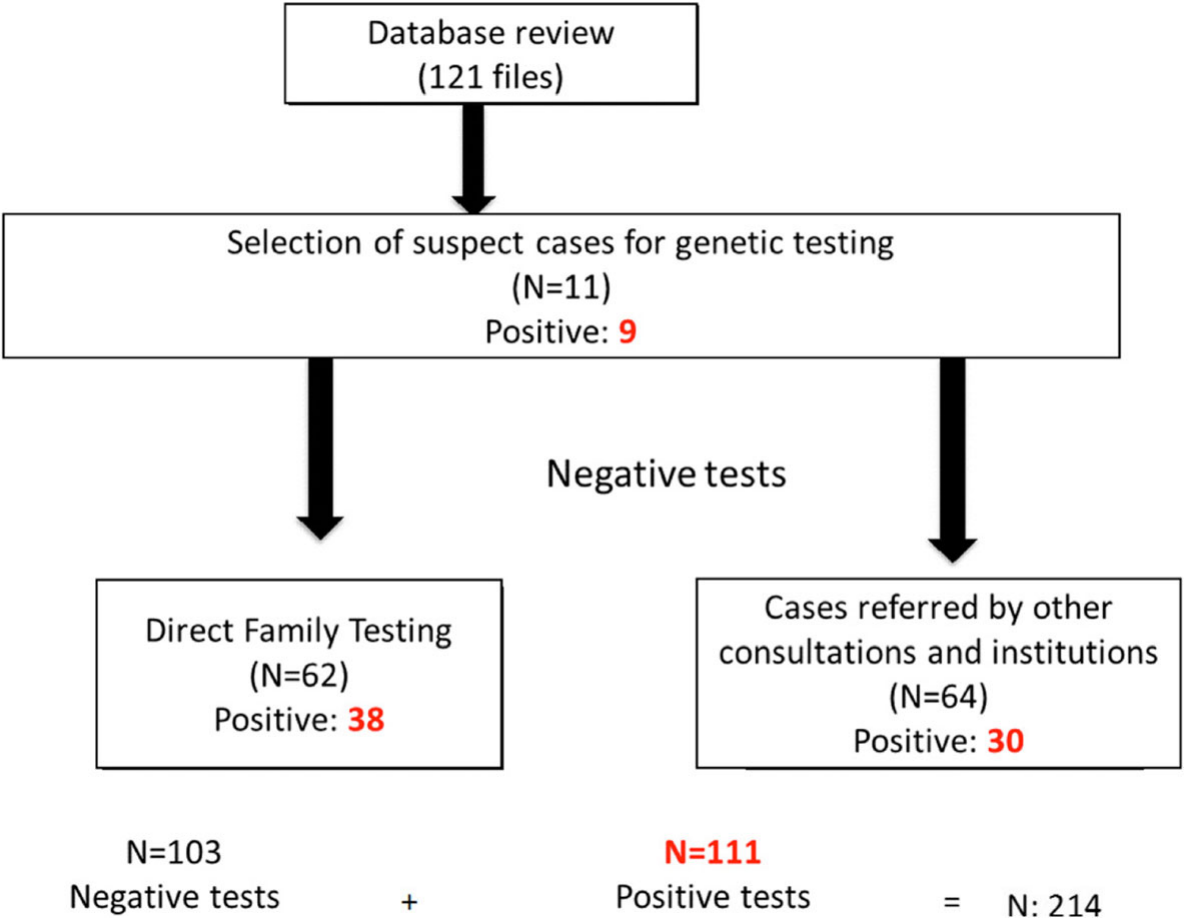
Design of the study. From 121 files, we selected 11 with possible hereditary amyloidosis, of which 9 were positive. Later, we screened 62 direct family relatives of the positive patients and 64 refered patients for testing. The total number of positive tests was 111

Eleven patients were chosen from a database of 121 files with a diagnosis of amyloidosis given their clinical characteristics. Nine (82%) of those pateints were positive for a TTR mutation. Sixty four patients were referred to our Center with the suspicion of hATTR for testing and 30 (46%) were positive. The remaining 62 patients were family relatives of patients with TTR mutations and 38 (61%) were positive. (Fig. 1).

### Demographic information

Of the patients positive for one of the TTR mutations, 55.3% were men and 44.7% were women. The mean age was 34.7 ± 13.1 years for women and 36.2 ± 11.3 years for men. At the time of the study, 23% patients were classified as asymptomatic carriers, 36% positive for mutation with disease-related symptoms, and 40% with positive mutation, characteristic symptoms and at least one positive biopsy for amyloid stained with Congo Red. Carriers and symptomatic patients without a positive biopsy for amyloid were much younger than symptomatic patients with positive biopsy.(23 vs 35 vs. 43 years of age, *p* = 0.000).

### Epidemiology

All the patients had a mestizo origin, the 4 grandparents of the index cases were born in Mexico and the patients did not present either Eastern or European traits.

### Place of origin

Forty (36%) patients were from Morelos, 46 (41%) from Guerrero, 12 (11%) from Mexico City, 12 (11%) from Guanajuato and one (0.9%) from another States of the Mexican Republic. (Fig. 1).

The main cities from which Guerrero patients came were Teloloapan, Cacalotenango, Oxtotitlan, Iguala, Taxco, Los Llanos, Coamazac and Acapulco. From the State of Morelos we had patients from Puente de Ixtla, Cuautla, Cuernavaca, Zacatepec, Xoxulta, Xiutepec and Zapata. Interestingly, the largest number of patients with mutations had an origin in Puente de Ixtla, a place that is located between the states of Morelos and Guerrero. All patients with the Ser52Pro mutation came from Guanajuato, in the town of San Luis de la Paz.

### Clinical presentation

Despite bearing different mutations, the patients presented similar clinical characteristics. The mean age at onset was 35.4 ± 11 years of age (range: 35-56). The initial symptom was neuropathic in 45 (40%) patients, gastrointestinal in 11 (10%), autonomic in 7 (6%) and cardiologic in 3 (3%). In the results of nerve conduction velocities, it was found that the nerves with the highest degree of affection both sensitive and motor, manifesting with a greater degree of affectation in the lower extremities in the sural nerve (59%) and the peroneal nerve (55%). The manifestations were earlier and more aggressive in men than in women.

### Family history

All patients except one have a pedigree where the segregation of the disease can be observed in at least 3 generations. In genealogies, however, the antecessor cases were not diagnosed by genetic testing due to lack of resources or by diagnostic errors, but the symptoms and the evolution of the disease were compatible with the diagnosis of amyloidosis. The age of presentation varied across different generations. In the first generation the average age was 53 years, in the second and third generations of 36 years, and in the fourth generation of 26 years, showing an anticipation phenomena.

### Outcome

Eleven patients (10%) died in the course of the study, with a mean age of 46 years, between 37 and 64 years-old, and duration of the disease since diagnosis between 1 and 4 years. Forty-five (41%) of the patients were asymptomatic at the time of the test, while 10% were in advanced stages according to the current classifications of the disease (Coutinho 1980, Yamamoto 2007) [11, 12]. Three patients received a liver transplant, of them, two are still alive and one died 1 year after the transplant. Two patients with severe biliary cirrhosis and liver adenocarcinoma received the livers of patients with amyloidosis, as a Domino transplant.

### Rough estimated prevalence in Mexico

During the 7 years of the study period, 111 positive tests were obtained for at least one TTR mutation in patient who came to the first consult in our Institution. To calculate the overall prevalence, we used the total number of patients who were seen in the first outpatient consult of all medical specialities from 2010 to 2017 in the Institution. The calculated prevalence (CP) is composed of the number of people affected (NPA) divided by the Total Number of Persons (TNP) can be represented graphically as follows:

$$ \mathrm{CP}=\mathrm{NPA}/\mathrm{TNP} $$

where:

NPA = Positive tests for at least one mutation of Amyloidosis by TTR mutation (*n* = 111).

*TNP = Total number of outpatients in the 1st. instead of attending INCMNSZ from 2010 to July 2017 (n = 157,686).*

Based on the above calculation, the estimated prevalence was 0.07% in the total population who had a first visit in our Institute. Considering that the total population in Mexico by 2017 is estimated at approximately 123.5 million inhabitants [13].^,^ Amyloidosis due to TTR mutations for Mexico would have a rate of 0.89 cases per 100,000. The result obtained within our institution and assumed for the population of Mexico is within the intervals estimated and published internationally by other countries that have studied these mutations in their own population, being the estimated prevalence in Japan the one that more is equated with that of Mexico. (Table 2). Table 3 compares the number of cases in relation to the surface area of the country, and total population, with other countries.

**Table 2:** Estimated Prevalence of Amyloidosis by TTR Mutations Worldwide

Country	Prevalence
Northern Portugal^a^	151/100,000
North of Sweden^a^	104/100,000
Mallorca, Spain^b^	5/100,000
Chipre^c^	3.72/100,000
Sicilia^d^	8.8/1,000,000
Japan^e^	0.87-1.1/ 100,000
México	0.89/100,000

^a^Parman, Curr Opin Neurol 2016, Planté-Bordeneuve, Lancet Neurol 2011

^b^Buades, Orphanet J Rare Dis 2014

^c^Dardiotis, Amyloid. 2019

^d^Mazzeo, J Neuromusc Dis 2015

^e^Kato-Motozaki Y J Neurol Sci 2008

**Table 3:** Prevalence between Mexico and other countries

Country	Total polulation (millions)	Surface area (thousands, km^2^	Number of diagnosed symptomatic TTR-FAP cases	Number of asymptomatic carriers of TTR gene mutation	Age range of patient cohort (years)
Portugal*	10.4	92.2	2000	> 500	18-87
Sweden*	9.6	438.6	250	Estimated 7500	25-85
France*	65.8	632.8	500	200	22-86
Itay*	60.8	302.1	500-600	250	25-85
Spain/ Majorca*	46.5	506	27	58	40-75
Bulgaria*	7.3	110	41	14	44-63
Germany*	80.5	357.3	120	60	28-69
Netherlands	16.8	41.5	45	23	25-75
Cyprus*	0.9	9.3	50	140	20-75
Turkey*	75	783.6	20-30	16	21-66
Japan**	127	378	110-135		37-78
Mexico	123.5	1964	85	26	18-77

*Adapted from the European Network for TTR-FAP (Parman, Curr Opin Neurol 2016)

**Data extracted from Kato-Motozaki Y J Neurol Sci 2008

**Fig. 2:**
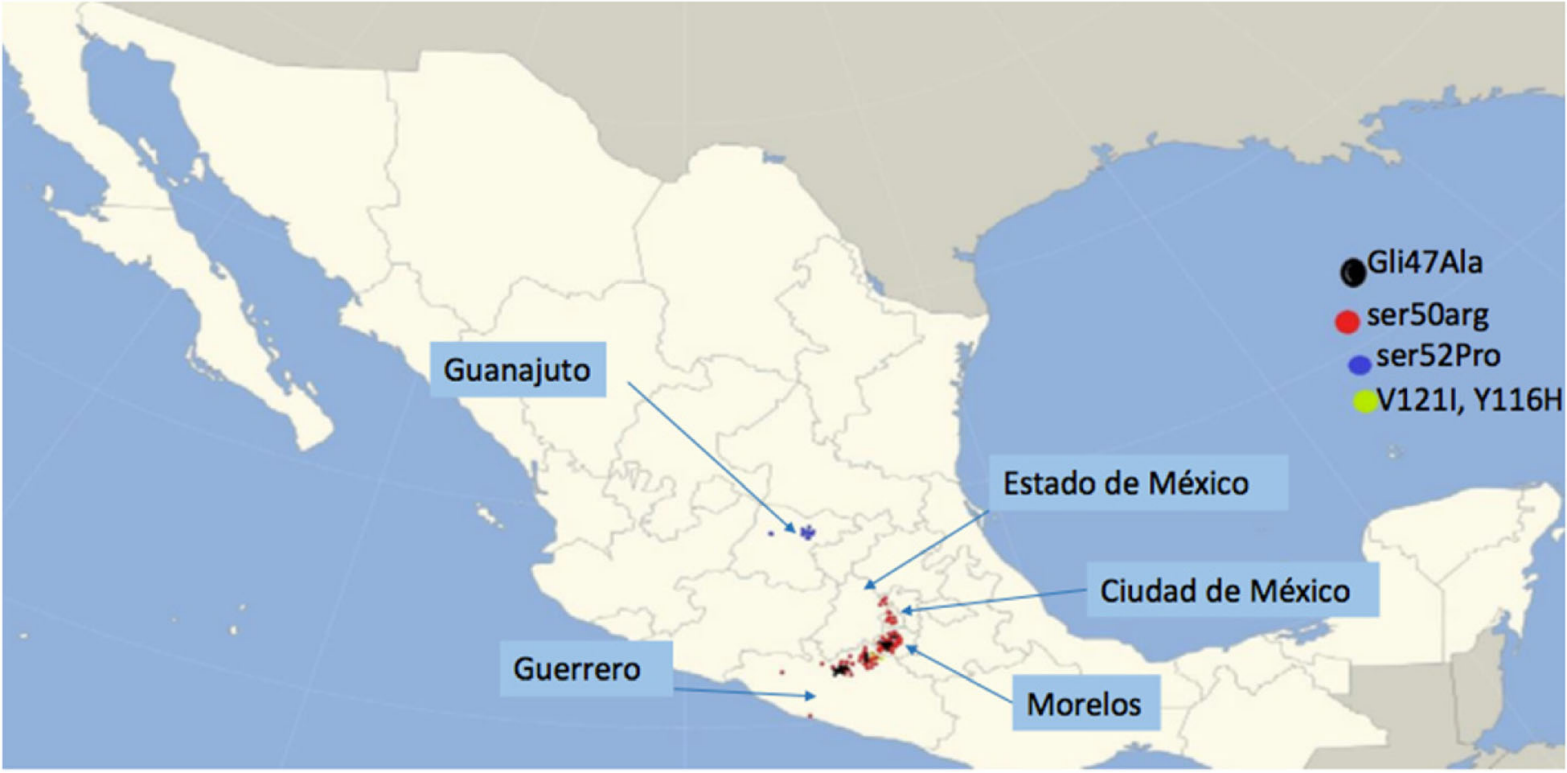
Epidemilogical map of the positive tests for at least one mutation of Amyloidsois by TTR mutations obtained in INCMNSZ. Geographical localization of hATTR cases in Mexico. Most of the cases were found following a trajectory from the Pacific Coast to the Center of the Country

Cases were clustered in two different geographical regions; the majority of cases came from Acapulco, the coast of the Pacific Ocean coast in Guerrero and followos a trail through Morelos to end in Mexico City (Fig. 2). This is similar to what happens in Spain, where a cluster is found in Majorca [14], and in Italy, where there is a big cluster in Sicily [15].

## Discussion

The present work describes five types of pathological mutations of the TTR gene in the Mexican population. Two things are relevant in our research: that patients are found mainly in two regions of the Mexican Republic: in the states of Guerrero and Morelos (mutations Ser50Arg, Gly47lAla and V122I /Y116H), with epicenter in Puente de Ixlta, and in Guanajuato (Ser52Pro mutation), and that no patient presented the Val30Met mutation, which is the most common mutation worldwide. The mutations we found have been well demonstrated as pathological and have also been described in Japan and Italy [1, 2, 16–18, 19–21],

Hereditary amyloidosis in Mexico was implicit for the first time in the study of Franco et al. [22] when reporting a cohort of 13 patients with a family history of amyloidosis and amyloid deposits in the eye; however, the type of mutation in the TTR gene or the origin of these patients was not determined at that time. In 2013 we described the clinical characteristics of some of these patients, however at that time we could not infer the epidemiology or its exponential growth [9, 10].

The founding effect refers to the installation of a population by a small number of individuals.[23, 24] Although the population may increase and become larger, the genes carried by all its members are derived from the few genes originally present in the founders. Random events affecting some genes present in the founders will have an important influence on the composition of the general population. When a population undergoes a drastic reduction in its size, it gives rise to a population with a high probability of presenting gene drift, that is to say, any mutation in an individual will be amplified in the community when it grows again [25]. Puente de Ixtla is a municipality of the State of Morelos that borders with the State of Guerrero, which has 56,410 inhabitants according to the last population census [26]. Originally tributary of the Aztecs, in the colonial era was a mandatory step for caravans merchants from Acapulco to the City of Mexico. During the Mexican Revolution it was evacuated almost in its totality [27]. It is possible that these population changes have laid the foundations for a founding effect that gave rise to a greater frequency of cases of amyloidosis in these States.

Genetic heterogeneity is a phenomenon where different mutations in the same locus cause the same phenotype, such as variants in the sickle cell gene, beta thalassemia in the beta globin gene, or Duchenne disease in the distrofin gene [27]. These allelic variations result from a process of natural selection, exogenous mutagens, genetic changes or genetic migrations. Many of these mutations may be in the form of a nucleotide polymorphism, where a single nucleotide base will be raised in comparison to a consensus sequence [27]. Alleles expressing allelic heterogeneity may be classified as adaptive or maladaptive [28].

The population we describe is similar to endemic populations (high numbers of patients in the same area) and different from sporadic hATTR-amyloid patients. The similarities are based on the following findings: 1) the onset of clinical manifestations is very early (35 years) compared to the amyloidosis reported in populations of sporadic origin where the disease presents patients over 50 years of age [1, 2]; 2) the picture is more aggressive in males than females, a prototypical characteristic of the endemic populations of Portugal [2]; and 3) clinical manifestations appear earlier in younger generations, a phenomenon that is also common only in endemic populations, somewhat similar to the anticipatory phenomenon seen in triplet diseases [27–34].

A major limitation of the study is the methodology we used, since it is not an open field study where a sampling of the population was taken, but rather an observational inference study, in a attempt to describe the presence of hereditary TTR amyloid mutations in Mexico, which up to this moment is ignored. Limitations are further depened because our Institution it is a referral center, therefore it is possible that the reference of patients is restricted to the Centers and Institutions that have some suspicion of a rare disease, as well as to the patients who can physically come to our Institution for their study. Also, incidence of positive TTR gene testing in relatives has its bias.

## Conclusions

In Mexico there are endemic foci of ATTR, mainly in the states of Morelos and Guerrero. The major mutations are different from the most common global mutation Met30Val. However, it is imperative to carry out larger studies aimed at determining the prevalence of familial amyloidosis in our country, in order to provide early genetic counseling and initiate timely treatment that improves the quality and life expectancy of patients with hATTR.

## Abbreviations

hATTR: Hereditary Amyloidosis TTR
TTR: Transthyretin
